# Continuous Immune Cell Differentiation Inferred From Single-Cell Measurements Following Allogeneic Stem Cell Transplantation

**DOI:** 10.3389/fmolb.2018.00081

**Published:** 2018-09-12

**Authors:** Yang Chen, Tadepally Lakshmikanth, Axel Olin, Jaromir Mikes, Mats Remberger, Petter Brodin

**Affiliations:** ^1^Unit of Clinical Pediatrics, Science for Life Laboratory, Department of Women's and Children's Health, Karolinska Institutet and Karolinska University Hospital, Stockholm, Sweden; ^2^Department of Oncology-Pathology, Karolinska Institutet, Stockholm, Sweden; ^3^Center for Allogeneic Stem Cell Transplantation, Karolinska University Hospital, Stockholm, Sweden; ^4^Department of Neonatology, Karolinska University Hospital, Stockholm, Sweden

**Keywords:** mass cytometry, CyTOF, stem cell transplantation, systems immunology, human immunology, diffusion maps

## Abstract

The process of immune system regeneration after allogeneic stem cell transplantation is slow, complex, and insufficiently understood. An entire immune system with all of its cell populations must regenerate from infused donor hematopoietic stem cells over the course of weeks and months post-transplantation. Both innate and adaptive arms of the immune system differ in their capacity and speed to reconstitiute in the recipient, which contributes to inadequacy in global immunity during the delayed reconstitution period. Systems-level analyses of immune systems in human patients have been made possible by high-throughput and high-dimensional, state-of-the-art, single-cell methodologies such as mass cytometry. Mass cytometry has revolutionized our ability to comprehensively profile all immune cell populations simultaneously in blood or tissue samples, providing signatures of differentially regulated cells in a range of clinical conditions. Such kind of systems immunology analyses promise not only for more accurate descriptions of variation between patients but also within individual patients over time, inter-dependencies between cell populations and the inference of developmental trajectories for specific cell populations. Here, we took advantage of a recently performed longitudinal mass cytometry analysis in 26 patients with hematological malignancies followed during the first 12 months following allogeneic stem cell transplantation. We present a proof-of-principle analysis to understand the evolution of individual immune cell populations. By applying non-linear dimensionality reduction and feauture extraction algorithms, we infer trajectories for individual immune cell populations, and map continuous marker expression changes occuring during immune cell regeneration that add novel information about this developmental process.

## Introduction

Human immune systems are highly variable between individuals and complex within individuals, consisting of multiple specialized cell populations that circulate and establish unique tissue niches. Fortunately, important information about even distal immune responses can be obtained from peripheral blood, the conduit of immune cells transiting from one organ to another (Brodin and Davis, 2016). Networks of such specialized cell populations give rise to immune responses and such responses can therefore only be fully understood if all participating immune cells are taken into account. Novel measurement techniques now allow for such simultaneous analyses of all cell populations present in the same blood sample (Davis et al., 2017). One such key measurement technique is mass cytometry (Bandura et al., 2009; Bendall et al., 2011). In mass cytometry, antibodies targeting proteins of interest are coupled to metal ions, each with a unique mass, and these can subsequently be detected using an ICP-MS instrument with single-cell resolution. Close to 50 different antibodies can now be combined and distinguished. This allows for the simultaneous analysis of all white blood cell populations in peripheral blood and variation in frequencies between individuals can be quantified (Brodin et al., 2015; Roederer et al., 2015; Carr et al., 2016). Using such novel assays, immune-mediated diseases and therapies aimed at modulating immune responses can be made more understandable (Kaczorowski et al., 2017). One example in recent years is the application of systems immunology analyses to vaccine-induced immune responses (Tsang et al., 2014; Hagan et al., 2015; Sobolev et al., 2016). Apart from predicting the strength of vaccine responses, information from these studies have revealed novel mechanisms of general importance to immunology at large (Ravindran et al., 2016). Also in tumor immunology, systems-level analyses are taking hold, providing novel information on the immune landscape of solid tumors (Chevrier et al., 2017; Lavin et al., 2017), and perturbations to immune cell networks associated with clinical outcome after allogeneic stem cell transplantation (Lakshmikanth et al., 2017). In the latter study, we analyzed immune cell and protein recovery during the first year after transplantation and identified differentially regulated features associated with clinical outcome of the patients (Lakshmikanth et al., 2017). Stem cell transplantation offers a unique opportunity for studying immune cell development and regeneration in humans, a process previously studied mostly in murine model systems (Davis and Brodin, 2018). Here we used this recently published mass cytometry dataset from patients undergoing allogeneic stem cell transplantation (Lakshmikanth et al., 2017) and performed a proof-of-principle analysis to reconstruct continuous immune cell development. We use diffusion maps to infer developmental trajectories for multiple immune cell populations and the results reveal novel aspects of immune cell regeneration after transplantation and improving our understanding of human immune cell differentiation and maturation after hematopoietic stem cell transplantation.

## Materials and methods

### Patients and blood samples

Twenty-six patients with hematological malignancies were involved in this study, who have undergone allogeneic stem cell transplantation at the Center for Allogeneic Stem Cell Transplantation (CAST), Karolinska University Hospital, between 2008 and 2013. Blood samples from these patients were drawn at 1, 2, 3, 6, and 12 months post-transplantation. Informed consent was provided by all patients in writing prior to their participation in accordance with the Declaration of Helsinki and our Ethical permit (2010/760-31/1) approved by the local Ethical review board in Stockholm.

### Mass cytometry analysis

PBMCs obtained by density gradient centrifugation from the blood samples of all patients were cryopreserved in freeze medium (90% FBS, 10% DMSO) and stored in liquid nitrogen. At the time of cell analyses, these cryopreserved PBMCs were thawed in RPMI medium supplemented with 10% fetal bovine serum (FBS), penicillin-streptomycin and benzonase (Sigma-Aldrich, Sweden). For live-dead cell distinction, cells were stained with 2.5 μM Cisplatin (Fluidigm) in RPMI without serum for 5 min at RT and quenched with RPMI containing 10% FBS. Cells were then re-suspended in CyFACS buffer (PBS with 0.1% BSA, 0.05% sodium azide and 2 mM EDTA), counted and around 1-2 million live cells were used for staining in a 96-deep well round bottom plate (Thermo Fisher Scientific, Waltham, MA), following which they were incubated for 30 min at 4°C with a 30 μl cocktail of metal conjugated antibodies against surface antigens. This was followed by a wash with CyFACS buffer and overnight fixation using 1% formaldehyde made in PBS (Polysciences Inc., PA, USA). For intracellular staining, cells were permeabilized with ice cold methanol (Sigma-Aldrich, Sweden) for 10 min at 4°C and stained with 30 μl of intracellular Ab cocktail (Ki-67) for 60 min at RT. Cells were washed and fixed in 4% formaldehyde at 4°C until acquisition. The antibody list is shown in Supplementary Table 1. Within a week after staining, cells were stained with DNA intercalator (0.125 μM Iridium-191/193 or MaxPar® Intercalator-Ir, Fluidigm) in 4% formaldehyde made in PBS for 20 min at RT. Cells were washed twice with CyFACS, once with PBS and milli-Q water, filtered through a 35 μm nylon mesh and diluted to 500,000 cells/ml. Cells were then acquired at a rate of 300–500 cells/s using a CyTOF 2 (Fluidigm) mass cytometer, CyTOF software version 6.0.626 with noise reduction, a lower convolution threshold of 200, event length limits of 10–150 pushes, a sigma value of 3 and flow rate of 0.045 ml/min.

### Antibodies and reagents

Antibodies for CyTOF analyses were obtained in a purified form formulated in carrier/protein-free buffer and were then coupled to lanthanide metals using the MaxPar X8 antibody conjugation kit (Fluidigm). The panel of antibodies is shown in Supplementary Table 1. The protein concentration was determined by measurement of absorbance at 280 nm, and the metal-labeled antibodies were diluted 1:1 in Candor PBS Antibody Stabilization solution (Candor Bioscience, Germany) for long-term storage at 4°C.

### Diffusion maps analysis

Manually classified populations (T-cells, B-cells, NK-cells, monocytes, and basophils) from one patient at a time, were merged across all time-points for calculation of diffusion maps using the destiny package within the Bioconductor release 3.2 (http://bioconductor.org/packages/release/bioc/html/destiny.html) used in R version 3.2.3 (2015-12-10) in R Studio server (version 0.99.473) on a 16 core Linux server (Centos 6.7). To identify changes associated with time, single-cell data was plotted colored by time-point and the diffusion components best describing transitions over time were identified.

## Results

### Cell population frequencies change over time after stem cell transplantation

In the context of immune system regeneration after transplantation, it is conceivable that immune cell phenotypes might exist that do not follow the expected canonical phenotypes. In order to improve the classification of five principal immune cell populations, we used a recently developed dimensionality reduction and manual clustering tool (ACCENSE, www.cellaccense.com) (Shekhar et al., 2014) to visualize single-cell level data in 2D (tSNE) and classify cell populations not based on individual markers, but on the basis of all 37 parameters analyzed (Supplementary Figure 1A). Using this method we selected T-, B-, and NK-cells, monocytes and basophils in each of the 26 patients at 1, 2, 3, 6, and 12 Months post-transplantation (Supplementary Figure 1A). Since relative frequencies fail to account for changes in total white blood cell counts and such changes are common and important aspects of immune system regeneration in this situation, we instead recalculated relative frequencies into absolute cell counts using clinical lab measurements of white blood cell counts and neutrophil counts (10^7^ cells/L of blood). This resulted in cell count measurements, which revealed drastic changes over time for each of these populations (Supplementary Figure 1B). T-cell counts increase up to about 150 days post-transplantation and plateau thereafter while B-cells engraft continuously, but slow-paced during the first year post-transplantation (Supplementary Figure 1B).

### Phenotypic changes revealed across cell populations at different time points after transplantation

**Figure 1:**
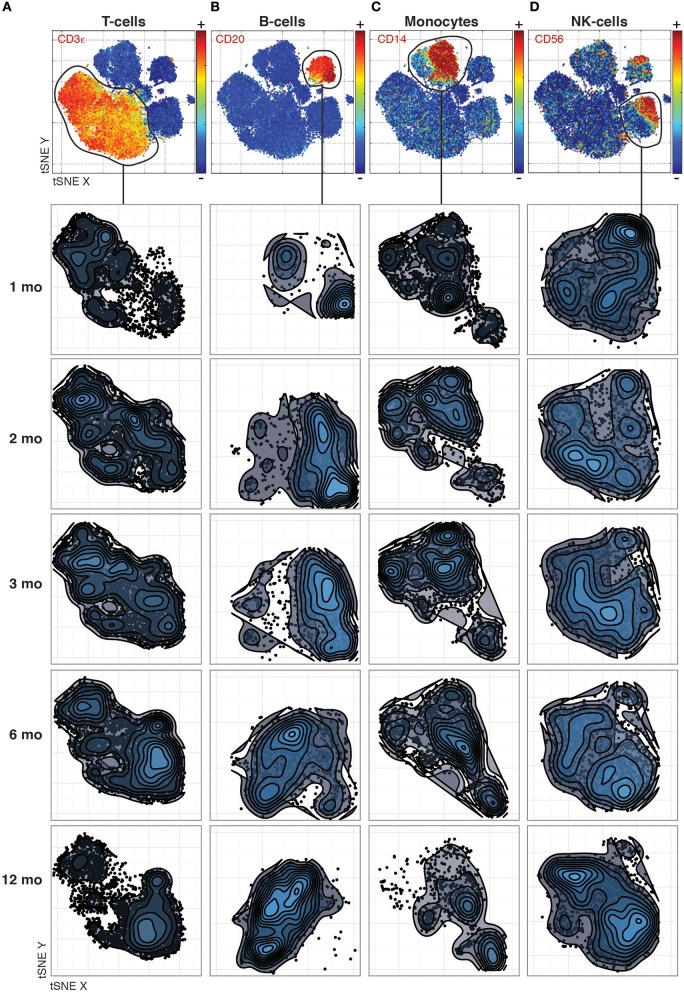
Phenotypic changes revealed across cell populations at different time points post transplantation. **(A)** T-cell phenotypes across time-points analyzed together by tSNE and visualized separately to make global structure of the data comparable across time-points. **(B)** B-cells, **(C)** Monocytes, and **(D)** NK-cells visualized as in **(A)**.

Even previously unknown cell populations which otherwise would be missed by manual gating based on expected marker combinations can be identified. The phenotypic distributions reveal striking changes between time-points that differ between the canonical cell populations. For T-cells, the most dramatic changes occurred between the 1st and 2nd month and between the 6th and 12th month (Figure 1A), while B-cell phenotypes changed completely between the 3rd and 6th month (Figure 1B). In contrast, monocyte phenotypes were largely stable throughout the year after transplantation (Figure 1C), while NK cell phenotypes were characterized by a convergence from multiple subpopulations into the two main subpopulations, CD56 bright and dim NK cells, by 12 Months after transplantation (Figure 1D).

### Diffusion maps reveal a continuous evolution of cell populations over time

**Figure 2:**
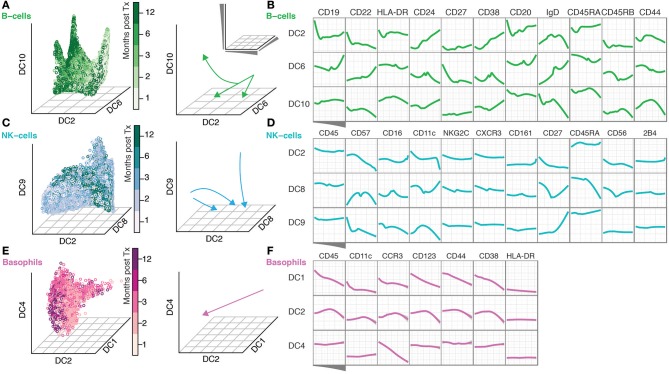
Diffusion Maps reveal a continuous evolution of cell populations over time. **(A)** Gated B-cells visualized along the time-variant DCs. Arrows indicate the direction of change over time. **(B)** The ordering of B-cells along the indicated DCs and median expression of the indicated markers. **(C,D)** NK-cells are shown as in **(A,B)**. **(E–F)** Basophils shown as in **(A,B)**.

The continuous nature of phenotypic changes occurring during immune reconstitution is difficult to appreciate from the discrete time-points alone. In order to reconstruct phenotypic trajectories during immune regeneration, we applied another non-linear dimensionality reduction algorithm, diffusion maps (Coifman et al., 2005; Haghverdi et al., 2015). When applied to mass cytometry data generated at discrete time-points, diffusion components, DCs (eigenvectors) that best correlate with time points, can be considered pseudo-time axes describing phenotypic evolution as a function of time after transplantation. In this way, marker expression changes along such pseudo-time axes reconstruct continuous phenotypic changes occurring after transplantation (Figure 2). B-cells showed a branched pattern of evolution along DCs 6, 2, and 10 that best correlated with time (Figure 2A). Changes in marker expression during the evolution of B-cells verify a previously described lack of CD27^+^ memory B-cells (Avanzini et al., 2005), but also reveal concomitant changes across a range of other markers (CD22, CD24, CD38, and CD44) (Figure 2B). NK cells evolve in a different pattern, starting out as a heterogeneous population and becoming increasingly focused with time into a more homogeneous one (Figure 2C). The convergence of NK cell phenotypes (toward the center of DC8) is accompanied by an increase in CD57, CD16 and a reduction in CD56 and CD27 expression (DC8 middle) (Figure 2D). In contrast, 2B4 and CD45 expression is stable during NK cell differentiation (Figure 2D). Basophils were relatively stable in frequency over the course of immune reconstitution (Supplementary Figure 1B), but nevertheless exhibited a clear phenotypic trajectory during immune reconstitution, most strongly represented by an inverse correlation with DC1 (Figure 2E). Along this trajectory, there is a continuous increase in canonical basophil markers CCR3, CD123, CD38, even for the more broadly expressed CD44 receptor (Figure 2F).

## Discussion

Canonical immune cell populations are typically defined based on prior knowledge of marker expression through a tedious process of manual gating. While biaxial plots showing two markers at a time are used to select cells with only a few markers taken into account to classify cells into specific cell populations, ACCENSE allows for visualization of cell data using 37 markers analyzed in a tSNE plot and this depicted the change in cell frequencies better in these patients analyzed post stem cell transplantation. Calculation of absolute cell counts revealed drastic changes overtime in cell populations. Early after transplantation, monocytes, and NK cells engraft and provide early protection from infectious disease complications (Storek et al., 2008). These dynamical changes reveal the complete reconfiguration of the immune systems in patients undergoing stem cell transplantation from an innate dominated system early to a more adaptive immune system later after transplantation.

Apart from these drastic changes observed, changes in phenotypes of cells add another layer of information needed to better understand the process of immune regeneration after transplantation. To investigate such changes in cell phenotypes in an unbiased manner, we took advantage of the dimensionality reduction and data visualization method, t-stochastic neighborhood embedding (tSNE) (van der Maaten and Hinton, 2008), previously applied also to mass cytometry data (Amir el et al., 2013). In the tSNE 2-d distribution plots, individual cells are aggregated into local neighborhoods together with other cells of similar phenotypes based not just on a few markers, but on the entire marker set analyzed (Supplementary Figure 1 and Figure 1).

These mass cytometry analyses reveal phenotypic changes occurring throughout regeneration albeit at different time intervals for different cell types, adding yet another layer of complexity to the process of immune cell reconstitution in patients undergoing stem cell transplantation. Together, our data illustrate that mass cytometry analyses performed at discrete time-points can be used together with diffusion maps to reconstruct continuous phenotypic trajectories during immune cell regeneration. To fully compare statistically, differences in the developmental processes of individual cell populations, more work will be required and new computational strategies devised.

## Author contributions

PB: Conception and study design; MR and PB: Sample collection; TL, AO, and PB: Mass cytometry panel development, optimization, and implementation; YC, PB, and TL: Data pre-processing, cell frequencies analysis, and cluster analysis (ACCENSE, Diffusion maps); YC and PB: Data visualization (tSNE); YC, TL, AO, JM, and PB: Manuscript preparation; YC, TL, AO, JM, MR, and PB: Critical reading and intellectual assessment of manuscript. All authors read and approved the final manuscript.

### Conflict of interest statement

YC, TL, AO, JM, and PB are founders and shareholders of Cytodelics AB (Stockholm, Sweden). The remaining authors declare that the research was conducted in the absence of any commercial or financial relationships that could be construed as a potential conflict of interest.

## Supplementary material

Supplementary Figure 1 Cell population frequencies changing over time after stem cell transplantation. **(A)** tSNE visualization of PBMCs at the indicated time-points post transplantation and colored by canonical cell population. **(B)** Changes in cell count for the indicated cell population over time after stem cell transplantation in all patients (*n* = 26).

Supplementary Table 1 Antibodies used in Mass cytometry experiments. Table indicates target marker name, clone ID, metal tag, and vendor for each antibody used.
