# Supplementary figures and images for: Continuous Immune Cell Differentiation Inferred From Single-Cell Measurements Following Allogeneic Stem Cell Transplantation
Source: Front Mol Biosci. 2018 Sep 12;5:81. doi: 10.3389/fmolb.2018.00081 (PMC6143687; doi:10.3389/fmolb.2018.00081)

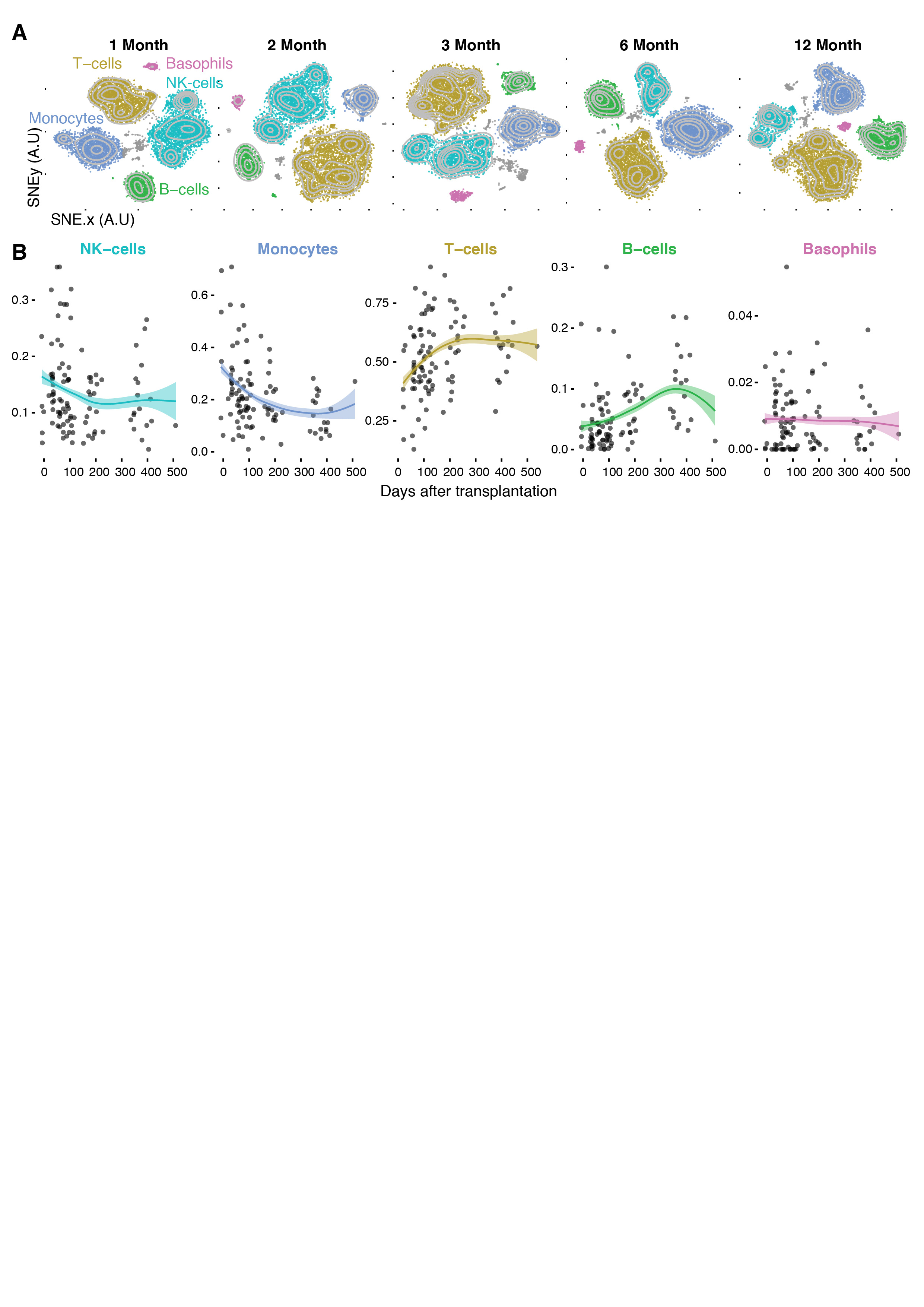

Supplement: Supplementary Figure 1 — Cell population frequencies changing over time after stem cell transplantation. (A) tSNE visualization of PBMCs at the indicated time-points post transplantation and colored by canonical cell population. (B) Changes in cell count for the indicated cell population over time after stem cell transplantation in all patients (n = 26). [file Image_1.JPEG]
